# Atad3 Function Is Essential for Early Post-Implantation Development in the Mouse

**DOI:** 10.1371/journal.pone.0054799

**Published:** 2013-01-25

**Authors:** Tobias Goller, Ursula K. Seibold, Elisabeth Kremmer, Wolfgang Voos, Waldemar Kolanus

**Affiliations:** 1 LIMES Institute, Program Unit Molecular Cell and Immune Biology, University of Bonn, Bonn, Germany; 2 Institute for Biochemistry and Molecular Biology, University of Bonn, Bonn, Germany; 3 Helmholtz Center Munich, German Research Center for Environmental Health, Institute for Molecular Immunology, Munich, Germany; Wellcome Trust Centre for Stem Cell Research, United Kingdom

## Abstract

The mitochondrial AAA+-ATPase ATAD3 is implicated in the regulation of mitochondrial and ER dynamics and was shown to be necessary for larval development in *Caenorhabditis elegans*. In order to elucidate the relevance of ATAD3 for mammalian development, the phenotype of an Atad3 deficient mouse line was analyzed. Atad3 deficient embryos die around embryonic day E7.5 due to growth retardation and a defective development of the trophoblast lineage immediately after implantation into the uterus. This indicates an essential function of Atad3 for the progression of the first steps of post-implantation development at a time point when mitochondrial biogenesis and ATP production by oxidative phosphorylation are required. Therefore, murine Atad3 plays an important role in the biogenesis of mitochondria in trophoblast stem cells and in differentiating trophoblasts. At the biochemical level, we report here that ATAD3 is present in five native mitochondrial protein complexes of different sizes, indicating complex roles of the protein in mitochondrial architecture and function.

## Introduction

ATAD3 belongs to the ancient family of AAA+-ATPases (ATPases associated with a wide variety of cellular activities) [1]. The protein structure of ATAD3 is characterized by two N-terminal coiled-coil domains, a central trans-membrane segment and a conserved C-terminal ATPase domain of the AAA+-type with an ATP-binding (Walker A motif) and a catalytic ATPase domain (Walker B motif) [2]. AAA+-ATPases are proposed to be chaperones or proteases and are involved in a variety of cellular processes e.g. cell cycle regulation, biogenesis of cell organelles and dis/assembly of protein complexes [3]–[5].

Except for humans, only one *ATAD3* gene locus is present in most species. During the development of the human lineage three genes, *ATAD3A*, *ATAD3B* [2] and *ATAD3C* [6], have likely been evolved by replication of a single precursor gene, as these three genes form a tandem array on chromosome 1.

A localization of ATAD3 to mitochondria was shown in several studies [2], [7], [8]. Analysis of ATAD3A topology in mitochondria by employing trypsin digestion experiments showed that the C-terminal AAA+-ATPase domain is located in the matrix, whereas a central trans-membrane segment anchors the protein in the inner membrane. The N-terminal domain interacts with the outer membrane [2]. It remains unclear, however, whether the N-terminus is spanning through the outer membrane into the cytosol. Nevertheless, an oligomerization of ATAD3A monomers has been proposed [2] which is supported by findings showing that other AAA+-proteins are assembling as hexameric rings [4], [9], [10].

It was proposed that ATAD3 is involved in the control of mitochondrial dynamics [2], [7]. Mitochondrial dynamics is mediated by fission and fusion of mitochondria, which are important for cell viability [11]–[14]. Also, the mitochondrial network gets fragmented during apoptosis resulting in smaller and more numerous mitochondria [15]–[18]. Down-regulation of ATAD-3 in *Caenorhabditis elegans* and in cultured human cells gave opposite effects on the mitochondrial network, respectively. Following RNAi of ATAD-3 in *Caenorhabditis elegans*, the mitochondria appeared thinner and slightly disorganized and the mitochondrial network was more filamentous [19]. In contrast, RNAi of ATAD3 in HeLa and lung cancer cells showed increased mitochondrial fragmentation and additionally a decreased co-localization of mitochondria and endoplasmatic reticulum (ER) [2], [6]. Mitochondrial fragmentation was also observed following overexpression of a Walker A deficient version of ATAD3A [2], [7]. Thus, ATP-bound ATAD3A might be required for the maintenance of mitochondrial integrity in mammalian cells [2].

Co-immunoprecipitation and two-dimensional immuno-blotting of mitochondria-associated membrane fractions revealed bindings of ATAD3A to the mitochondrial fission protein dynamin-related protein 1 (DRP1) and to the mitochondrial fusion proteins mitofusin-2 (MFN2) and optic atrophy 1 (OPA1) [6]. DRP1 is a GTPase that is normally located to the cytoplasm, but is recruited to the mitochondria by binding to its receptor Fis1, which resides in the outer mitochondrial membrane [20], [21]. DRP1 mediates fission of mitochondria by formation of a homo-multimeric complex [22]–[24]. MFN2 and the structurally related mitofusin-1 (MFN1) are GTPases, which are located in the outer mitochondrial membrane [25]–[28]. A fraction of MFN2 is also present in the membrane of the ER, where it mediates the contact between ER and mitochondria by homotypic (MFN2-MFN2) and/or heterotypic (MFN2-MFN1) binding [29]–[31]. The GTPase OPA1 is incorporated into the inner mitochondrial membrane, where it mediates fusion of the inner membrane and christae formation [28], [32]–[34]. It was shown that YME1L1 and m-AAA protease, members of the family of AAA+-ATPases regulate OPA1 processing and mitochondrial fusion [35], [36]. An interaction of ATAD3A with the apoptosis inducing factor AIF was proposed [6]. During apoptosis AIF is released from the mitochondrial inter-membrane space and locates to the nucleus where it interacts with histone H2AX and promotes chromatinolysis [37], [38]. Additionally, an interaction between ATAD3A and calcium-binding protein S100B was shown [39]. Finally, ATAD3A bindings to the D-loop of the mitochondrial DNA (mtDNA) molecule and to ribosomes, and its involvements in the regulation of mtDNA replication, transcription of mtDNA encoded genes and mitochondrial protein synthesis were discussed [40]–[44].

An essential role of ATAD3 for development was demonstrated in *Caenorhabditis elegans*, because RNAi of ATAD-3 in the worm system causes severe defects, characterized by early larval arrest, gonadal dysfunction and embryonic lethality [19].

To date analyses of the localization and the function of ATAD3 family members were performed in human cell lines or in *Caenorhabditis elegans*. Until now there is no genetic evidence for the relevance of ATAD3 function in mammalian development or disease. In this article the early post-implantation phenotype of an Atad3 loss-of-function mutation in the mouse is described. Furthermore we analyzed the contribution of ATAD3 to the formation of native mitochondrial protein complexes.

## Results

### In the Mouse Two Atad3 Protein Isoforms are Generated from a Single Gene by Alternative Splicing

**Figure 1:**
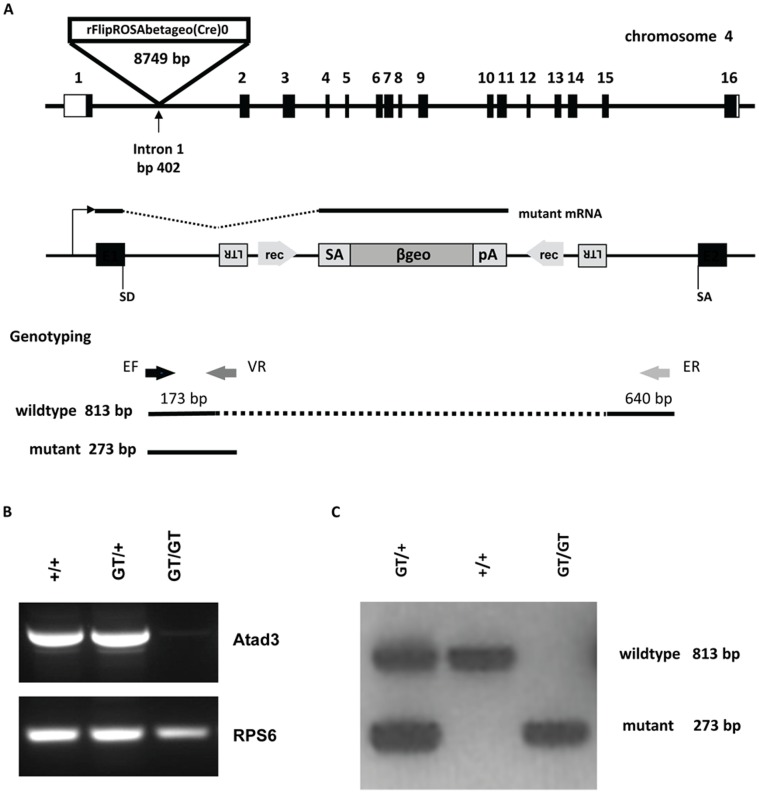
Gene trap mutagenesis of the murine *Atad3* locus. **A** Schemes of the murine *Atad3* genomic locus on chromosome 4 showing the integration site of the gene trap vector rFlipROSAbetageo(Cre)0 into intron 1 at position 402 as an overview (upper scheme) and in detail (middle scheme), as well as the positions of the genotyping primers and resulting amplificates (lower scheme). Boxes in the upper scheme represent all 16 exons, the untranslated regions (UTRs) are without filling. In the middle scheme it is depicted, that the gene trap vector insertion leads to a fusion of 5? exon 1 (E1) to the splice acceptor side (SA) of rFlipROSAbetageo(Cre)0. The mutant mRNA contains *Atad3* exon 1 and the vector *βgeo* cassette and is terminated by an SV40 polyadenylation signal (pA). Retroviral long terminal repeat (LTR) sequences are necessary for efficient vector integration. Recognition sites (rec) for Flp and Cre recombination enzymes allow first the reconstitution of the wildtype mRNA and secondly a subsequent conditional mutagenesis. In the lower scheme, arrows indicate the positions of the PCR primers EF (endogenous intron 1 forward primer), ER (endogenous intron 1 reverse primer) and VR (gene trap vector reverse primer) used for genotyping. Constitution of wildtype (813 bp) and mutant (273 bp) PCR fragments are shown below. **B** RT-PCR analysis of E6.5 embryonic samples shows that the complete 3? region (exons 11 to 16) of the *Atad3* cDNA is missing in *Atad3^GT/GT^* embryos, instead in wildtype and *Atad3^G/+^* tissues a 805 bp fragment represents the *Atad3* cDNA. Amplification of ribosomal protein S6 (RPS6) cDNA serves as quantity control. **C** For genotyping of the *Atad3* locus, wildtype and mutant genomic PCR amplificates are separated in a 1.5% agarose gel and identify samples from wildtype (*+/+*), heterozygous (*GT/+*) and homozygous mutant (*GT/GT*) individuals.

In murine mRNA and protein databases (NCBI, Ensembl), two Atad3 isoforms are annotated, which are derived from a single gene by alternative splicing. Isoform 1 is referred as the full length Atad3 cDNA of 2412 bp with an open reading frame of 1776 bp encoding for a protein composed of 591 amino acids and with a molecular weight of 66.742 kDa. Alignment of mouse Atad3 isoform 1 cDNAs (accession numbers NM_179203 and BC058373) and the mouse genomic sequence (accession number NT039268) by BLAST (Basic Local Alignment Search Tool) indicates that the murine *Atad3* gene is located on chromosome 4 and is composed of 16 exons, extending over a genomic locus of around 20.5 kb (Fig. 1A). Additionally a highly (96%) homologous sequence of similar length (2316 bp) is located on chromosome 15. This sequence likely is thought to be a pseudo-gene as it is typically composed of only a single exon-like section without any intronic interruptions. Atad3 isoform 2 encodes a shorter protein of 512 amino acids and a molecular weight of 57 kDa. Isoform 2 is generated by alternative splicing of exons 13 and 14, which leads to a subsequent translational frame shift. The murine Atad3 protein isoform 1 shows an identity of 92.1% in its amino acid sequence to the human orthologue ATAD3A (NP_001164007) which has a molecular weight of 66 kDa. Both murine isoforms contain two N-terminal coiled-coil domains, central trans-membrane segments, and Walker A and Walker B motifs, respectively. Interestingly, the C-terminal portion of the AAA+-ATPase domain, directly positioned after the Walker B motif in isoform 1, is missing in isoform 2.

### Gene Trap Disruption of the Murine *Atad3* Gene Leads to a Loss-of-function Mutation

The E14TG2a.4 (129SV2) ES cell clone E118D03 (offered by the German Gene Trap Consortium) carrying a gene trap mutation in one *Atad3* allele (*Atad3^GT^*) was used to establish a stable mouse line that had transmitted the mutation into the germ line. In this ES cell clone, the gene trap vector rFlipROSAbetageo(Cre)0 is integrated after nt 402 into the first intron of the *Atad3* gene, generating a fusion transcript by splicing *Atad3* exon 1 at its splice donor site (SD) to the splice acceptor site (SA) of a transgenic cassette (*βgeo*), which encodes for the bacterial LacZ reporter gene and a neomycin phosphotransferase selection marker. Termination of transcription is mediated by a SV40 polyadenylation signal (Fig. 1A). A RT-PCR approach for amplification of a sequence containing exons 11 to 16 failed to detect a cDNA transcript, which proves that the described gene trap event in the *Atad3* locus leads to a complete loss of the 3?encoded region in *Atad3^GT/GT^* tissues (Fig. 1B) and therefore represents a loss-of-function mutation. The resulting fusion protein contains only the first 67 amino acids of the wildtype Atad3 protein, i.e. the N-terminal part of the first coiled-coil domain. As the trans-membrane and the AAA+-ATPase domain are completely missing, the mutant protein is rendered dysfunctional.

Genotyping of mice and embryos was performed by PCR, employing three primers. The wildtype allele is represented by an 813 bp long fragment, whereas the mutant allele (*Atad3^GT^*) is characterized by a 273 bp fragment of endogenous and transgenic origin (Fig. 1A, C).

### *Atad3^GT/GT^* Embryos Exhibit Retarded Post-implantation Development and Die Around E7.5

**Figure 2:**
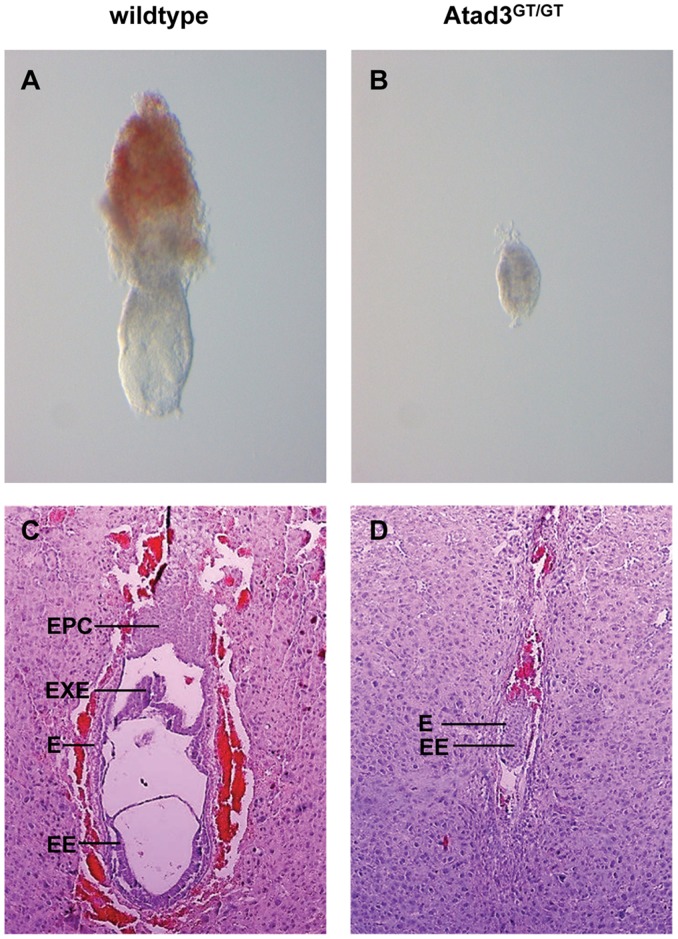
*Atad3^GT/GT^* embryos are defective in early post-implantation development. **A, B** Morphology of a wildtype (A) and an *Atad3^GT/GT^* (B) embryo at E6.5. *Atad3^GT/GT^* embryos show a total growth reduction, especially the proximo-distal axis is not extended. **C, D** Anatomical analysis of a wildtype (C) and an *Atad3^GT/GT^* (D) embryo at E7.5 by hematoxilin/eosin staining of 7 µm paraffin sections (same magnifications). In *Atad3^GT/GT^* embryos EPC and EXE are strongly reduced. Additionally, EE and E appear less differentiated. Abbreviations: E endoderm, EE embryonic ectoderm, EPC ectoplacental cone, EXE extra-embryonic ectoderm.

Genotyping showed that heterozygous Atad3 (*Atad3^GT/+^*) mice are viable and fertile. *Atad3^GT/+^* mice exhibit no obvious phenotype. When offspring from heterozygous parents was genotyped, no homozygous mutants (*Atad3^GT/GT^*) were obtained, suggesting that the mutation results in recessive lethality during embryonic development. Indeed, the genotype distribution of embryos obtained from heterozygous intercrosses which were isolated between the stages E6.5 to E8.5 reveals that *Atad3^GT/GT^* embryos die before E8.5. Between E6.5 and E8.5 the ratio of vital *Atad3^GT/GT^* individuals decreases from 20.6% to 0.0%, whereas the ratio of detectable resorptions increases markedly from 5.9% to 32.9% (Table 1). Because of the complete degradation of the respective embryonic tissues, resorptions were not genotyped. Detectable numbers of *Atad3^GT/GT^* embryos and resorptions at the analyzed embryonic stages are found to be close to the expected Mendelian ratio of 25%. All *Atad3^GT/GT^* embryos are developmentally retarded and show the same abnormal morphology. The phenotype is characterized by a low variability in size and morphology of the mutant embryos at E6.5 (n >14) and E7.5 (n >12) and a constant time point of lethality between E7.5 and E8.5. Compared to wildtype embryos at the egg cylinder stage E6.5 (Fig. 2A), *Atad3^GT/GT^* embryos show a total growth reduction, have an oval to conic shape, and specifically the proximo-distal axis is not extended (Fig. 2B). Furthermore, the ectoplacental cone, marked by its red colour is not visible in *Atad3^GT/GT^* embryos, indicating that the differentiation of extra-embryonic tissue is disturbed and reduced (Fig. 2B). As the overall growth of murine embryos is minimal between E5.5 and E7.5, only an embryo of the final vital stage E7.5 is depicted in Figure 2B. Histological analysis gives a more precise view on the developmental retardation of *Atad3^GT/GT^* embryos. Along their proximo-distal axis, wildtype egg cylinder stage embryos have developed three tissues, which are the embryonic ectoderm, the extra-embryonic ectoderm and the ectoplacental cone (Fig. 2C). Embryonic ectoderm and extra-embryonic ectoderm are surrounded by the endoderm. In contrast, *Atad3^GT/GT^* embryos (n = 3) at the gastrula stage (E7.5) resemble wildtype embryos of the stage E5.5, because internal cavitation is completely missing. The ectoplacental cone and also the extra-embryonic ectoderm are at least strongly reduced, maybe even completely absent. Additionally, the embryonic ectoderm and endoderm appear less differentiated (Fig. 2D). Absence of a proamniotic canal clearly indicates that the development of the embryonic ectoderm is also affected by the mutation. But since firstly, the effect of the mutation appears to be more dramatic on the formation and differentiation of extra-embryonic tissues, and since secondly, the extra-embryonic tissue is known to have a strong influence on the proximo-distal growth and survival of the complete embryo during early gastrulation, further analyses were focused on the importance of Atad3 function on trophoblast development.

**Table 1:** Lethality of Atad3 deficient mice.

Stage	Individuals	Genotype			
		*+/+*	*GT/+*	*GT/GT*	resorbed
**E6.5**	68 (8 litters = >8.5/litter)	14 (20.6%)	36 (52.9%)	14 (20.6%)	4 (5.9%)
**E7.5**	76 (8 litters = >9.5/litter)	13 (17.1%)	43 (56.6%)	12 (15.8%)	8 (10.5%)
**E8.5**	73 (9 litters = >8.1/litter)	13 (17.8%)	36 (49.3%)	0 (0.0%)	24 (32.9%)

### *In vitro* Cultured *Atad3^GT/GT^* Embryos Only Form a Minimal Trophoblast Outgrowth

**Figure 3:**
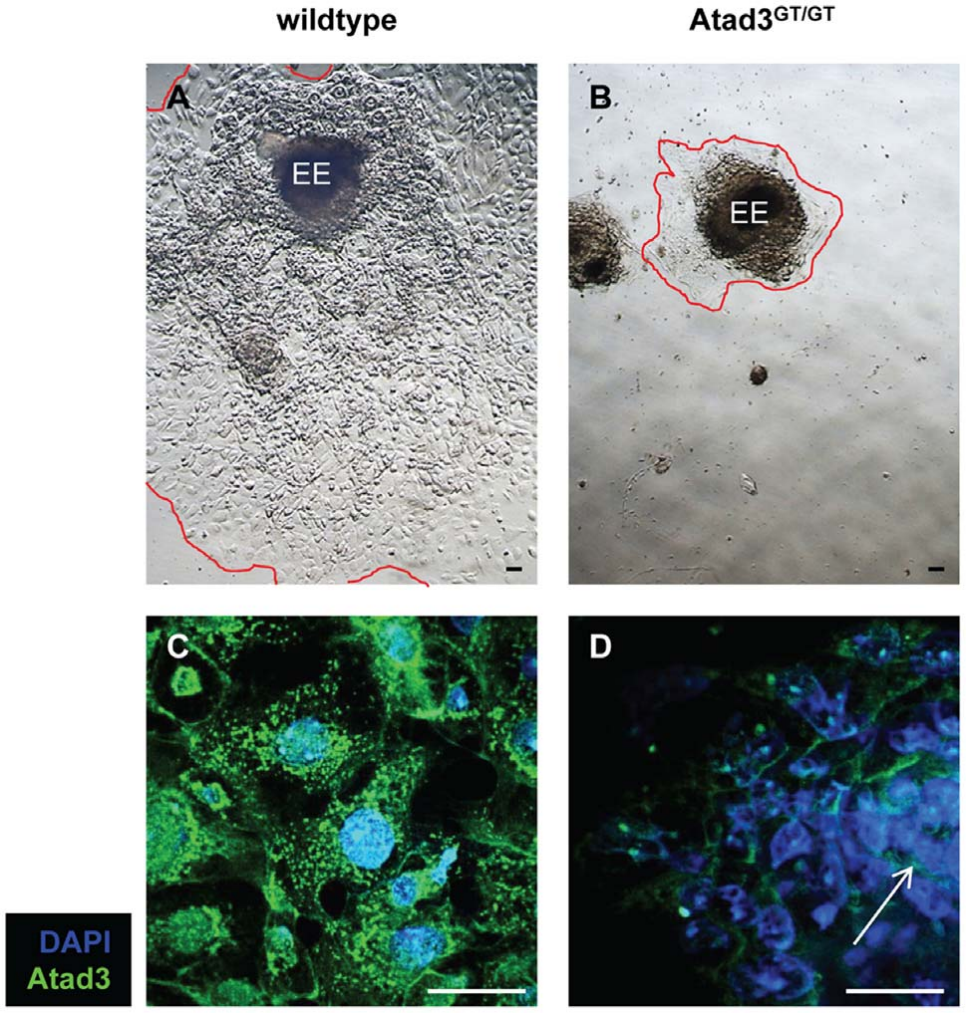
*Atad3^GT/GT^* embryos are defective in trophoblast development. **A, B** Morphology of a wildtype (A) and an *Atad3^GT/GT^* (B) embryo outgrowth, isolated at E6.5, and after 3 days of *in?vitro* cultivation. Red lines mark the borders of the outgrowths. Compared to the wildtype tissues, the size of the epiblast remains clearly diminished in *Atad3^GT/GT^* embryos. EE epiblast.**C**, **D** Atad3 protein expression and mitochondrial morphology in the middle region of a wildtype trophoblast outgrowth (C) and a complete *Atad3^GT/GT^* (D) embryo outgrowth are shown by immuno-staining with an Atad3 specific antibody (green). Nuclei are visualized by DAPI staining (blue). In *Atad3^GT/GT^* outgrowths, Atad3 protein is only weakly expressed in the epiblasts and is almost undetectable in the trophoblast. The white arrow in D indicates the epiblast.

To investigate the effect of Atad3 deficiency on the formation and function of extra-embryonic tissue on the subcellular level, mitochondrial morphology and trophoblast differentiation was analyzed in outgrowths of E6.5 embryos, *in?vitro*. Cultivated post-implantation embryos continue with the imminent steps of the developmental program. In wildtype embryos of this stage (n = 26), trophoblast derived tissue grows out radially from an already existing small ectoplacental cone, and forms more differentiated cell types and finally polyploid giant cells at the leading edge (Fig. 3A). Although attachment to the gelatine covered dish is mainly mediated by trophoblast tissue, most *Atad3^GT/GT^* embryos (n = 9) are able to adhere indicating the existence of minimal tissue emerging from the polar trophectoderm. Compared to the wildtype tissues, the size of the epiblast remains clearly diminished in *Atad3^GT/GT^* embryos even after three days of culture (Fig. 3B). During three to four days of culture, *Atad3^GT/GT^* embryos form only minimal outgrowths (Fig. 3B). The variability in sizes of the outgrowths is up to 50%. In wildtype embryos, immuno-staining reveals a moderate amount of Atad3 protein in both the epiblast and the trophoblast (Fig. 3C). In contrast, Atad3 protein is only weakly expressed in the epiblasts of *Atad3^GT/GT^* outgrowths and is almost undetectable in the trophoblast (Fig. 3D). Existence of Atad3 protein in the mutant epiblast might be due to persisting protein contribution of the oocyte throughout pre-implantation development until the early post-implantation stages.

### Mitochondrial Morphology is Modified during Trophoblast Development

**Figure 4:**
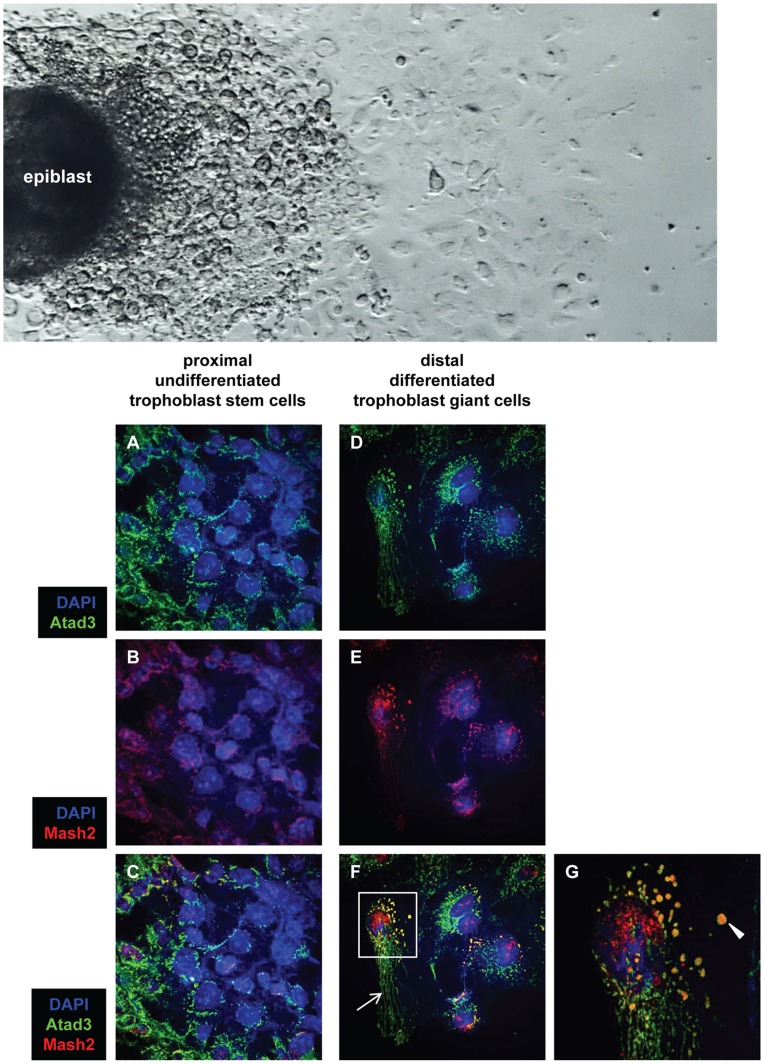
Alterations in mitochondrial morphology during trophoblast differentiation. The overview picture shows the different areas of a typical E6.5 embryo outgrowth (outgrowth direction from left to right): the epiblast mountain (left), followed by the proximal zone of densely arranged undifferentiated trophoblast stem cells and finally the distal zone (right) of differentiated trophoblast cells. **A–G** The alteration of mitochondrial morphology during proximo-distal trophoblast differentiation in wildtype outgrowths is shown by co-immunostaining of the mitochondrial marker Atad3 (green) and the trophoblast differentiation marker Mash2 (red). Nuclei are visualized by DAPI staining (blue). In cells of the proximal trophoblast (A – C), the mitochondria are small and diffusely distributed (A), here Mash2 is localized to the cytoplasm (B). Instead in the distal region of the outgrowths (D – G), the cells contain enlarged, swollen mitochondria (D, F and arrowhead in G), and additionally small mitochondria arranged in arrays from the center to the periphery of the cell (D, arrow in F). In distal cells, Mash2 is localized to the nucleus and is also detected in the matrix of the swollen mitochondria (E, F and G). G is a magnified detail of F.

The modification of the mitochondrial morphology and the mitochondrial network during trophoblast differentiation was studied by co-immunostaining for Atad3 and Mash2 (mouse achaete-scute homolog 2) in wildtype outgrowths (n = 13). Atad3 and its orthologues in other species (*Homo sapiens*, *Drosophila melanogaster*) are excellent trackers for mitochondrial populations in cells, as they are abundantly expressed in this organelle and span the inner membrane, the inter-membrane space and probably also the outer membrane [2]. Differentiation of the trophoblast lineage from the proximity of the center to the distal area of the outgrowth is characterized by the expression and nuclear import of the bHLH transcription factor Mash2, which indicates differentiated trophoblasts and trophoblast giant cells. In cells of the proximal trophoblast, where Mash2 protein is localized to the cytoplasm (Fig. 4B), the mitochondria are small and diffusely distributed (Fig. 4A, C). In the distal zone of the outgrowths the cells contain enlarged, swollen mitochondria (Fig. 4D, F, G), and additionally small mitochondria arranged in arrays from the center to the periphery of the cell (Fig. 4D, F). In these cells, Mash2 protein has translocated into the nucleus as expected (Fig. 4E–G), but surprisingly is also detected in the matrix of the swollen mitochondria (Fig. 4G).

### *Atad3^GT/GT^* Embryos are Defective in Trophoblast Stem Cell Maintenance and Trophoblast Differentiation

**Figure 5:**
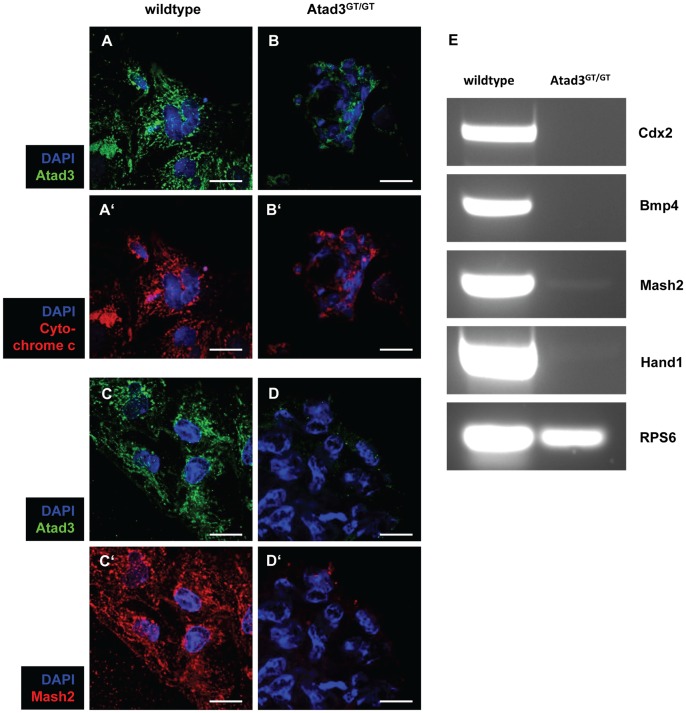
*Atad3^GT/GT^* embryos are defective in trophoblast differentiation. **A**, **B** Intrinsic apoptosis in wildtype (A) and *Atad3^GT/GT^* (B) embryo outgrowths at E6.5 was analyzed by immuno-staining for cytochrome c (red). Mitochondria in wildtype (A) and *Atad3^GT/GT^* (B) cells are marked by Atad3 immuno-staining (green). A punctate cytochrome c expression pattern is seen in wildtype (A’) as well as in *Atad3^GT/GT^* cells (B’). **C**, **D** Differentiated trophoblast cells in in the most distal region of wildtype (C) and *Atad3^GT/GT^* (D) embryo outgrowths at E6.5 were detected by immuno-staining for Mash2 (red). Mitochondria in wildtype (C) and *Atad3^GT/GT^* (D) cells are marked by Atad3 immuno-staining (green). Analysis was performed by confocal microscopy. In contrast to wildtype cells (C’), Mash2 is rarely detectable in cells of the most distal region of the *Atad3^GT/GT^* trophoblast outgrowth (D’). All nuclei are visualized by DAPI staining (blue). **E** The expression of trophoblast cell type specific marker genes in E6.5 wildtype and *Atad3^GT/GT^* embryos was monitored by RT-PCR. As opposed to strong signals for all analysed markers in the wildtype sample, in the *Atad3^GT/GT^* specimen Cdx2 and Bmp4 transcripts are not detected and Mash2 and Hand1 amplificates are observed at very low levels.

Next we wanted to elucidate whether Atad3 deficiency had an effect on mitochondrial morphogenesis, intrinsic apoptosis and differentiation of the trophoblast. If cell death were the reason for the reduction of trophoblast size, intrinsic apoptosis might be expected, which is characterized by the release of cytochrome c from mitochondria to the cytoplasm. In wildtype cells (n = 8) the punctate cytochrome c expression as well as the explicit co-localization with the mitochondrial tracker Atad3 indicates that intrinsic apoptosis does normally not occur in the trophoblast (Fig. 5A’). In *Atad3^GT/GT^* cells (n = 3), a robust punctate cytochrome c pattern is also apparent, indicating that intrinsic apoptosis in the mutant trophoblast can be excluded (Fig. 5B’). Additionally, the cytochrome c expression pattern itself highlights regularly formed mitochondria in the few cells, of which the *Atad3^GT/GT^* trophoblast consists. Fragmentation of the nuclei is a general hallmark for apoptosis. But neither in wildtype (n = 26) nor in *Atad3^GT/GT^* embryos (n = 9), DAPI staining reveals any fragmented nuclei. Since accelerated apoptosis could be ruled out, the competence of Atad3-defective trophoblast stem cells to differentiate into mature trophoblast cells was monitored by immuno-staining for the differentiation marker Mash2. And indeed, Mash2 is rarely detectable in cells of the complete *Atad3^GT/GT^* trophoblast outgrowth (n = 13) (Fig. 5D’) as compared to wildtype cells (n = 3) (Fig. 5C’), proving the disability of trophoblast stem cells or their very early descendants to differentiate into later trophoblast cell types or trophoblast giant cells. Additionally, the expression of several marker genes for trophoblast stem cells (Cdx2), extra-embryonic ectoderm (Bmp4) and ectoplacental cone (Mash2, Hand1) in wildtype (n = 3 pools of minimum 4 embryos) and *Atad3^GT/GT^* embryos (n = 3 pools of minimum 4 embryos) of the stage E6.5 was monitored by RT-PCR (Fig. 5E). Interestingly, Cdx2 and Bmp4 mRNAs are not verifiable at all. Likewise, Mash2 and Hand1 transcripts are rarely traceable in *Atad3^GT/GT^* embryos. This indicates that not only the differentiation within the early trophoblast is impaired, but already the maintenance of the trophoblast stem cell pool is affected in *Atad3^GT/GT^* embryos.

### ATAD3 Contributes to Five Mitochondrial Protein Complexes of Different Molecular Weight

**Figure 6:**
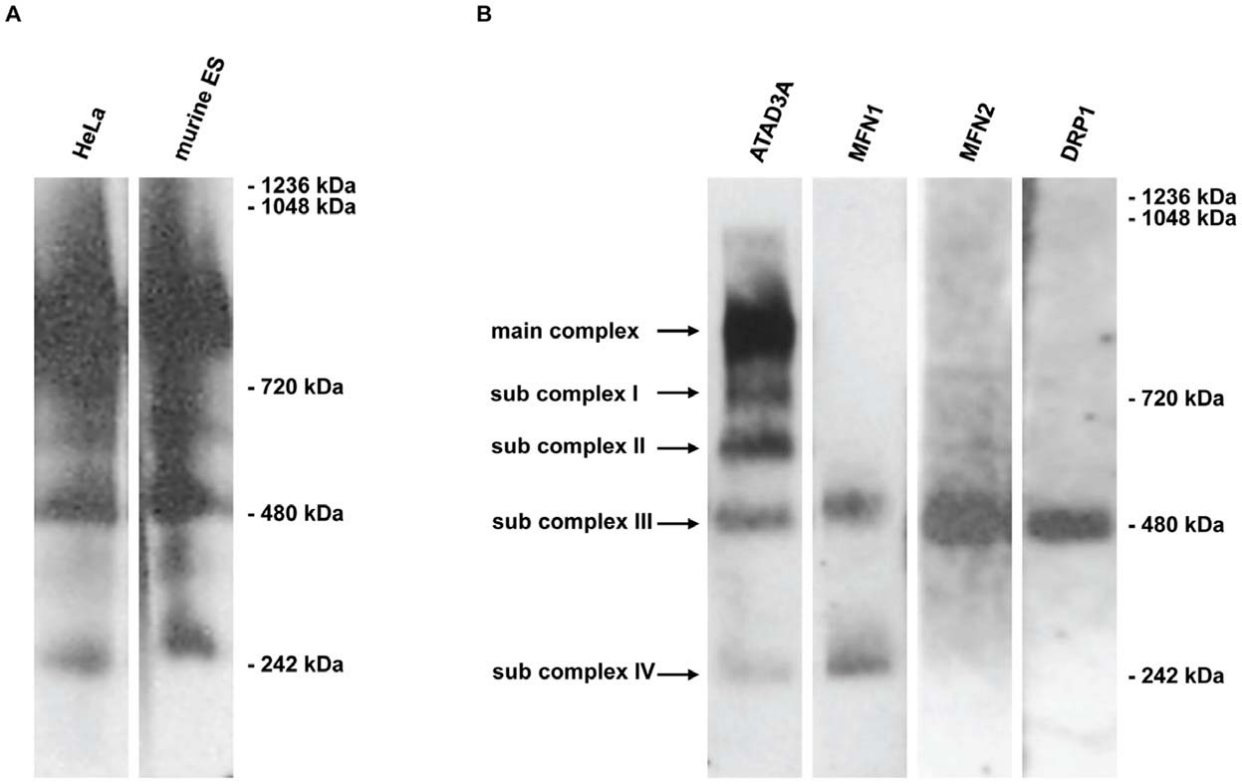
ATAD3 contributes to five mitochondrial protein complexes of different sizes. **A** The expression of human ATAD3A/Atad3 in different mitochondrial protein complexes in HeLa cells (first lane) and in the murine ES cells (second lane) is shown by Blue native PAGE (gradient of acrylamide concentration: 3–12%) and subsequent immuno-blotting for ATAD3A. In both cell lines the same expression pattern is seen. Both lanes depicted have run on the same gel and are taken from the same ATAD3A immuno-blot. **B** In HeLa cells, ATAD3A is detected in five different protein complexes (first lane). The largest and most abundant complex (main complex) has a molecular weight of about 800 to 900 kDa. The four smaller sub-complexes I–IV have estimated molecular weights of about 720, 600, 480 and 240 kDa. In HeLa cells, Blue native PAGE (gradient of acrylamide concentration: 3–12%) and subsequent immuno-blotting shows that the mitochondrial fission and fusion proteins MFN1, MFN2 and DRP1 are contained in protein complexes of similar molecular weights as ATAD3A sub-complexes III and IV (second to fourth lane). All four lanes depicted have run on the same gel. Sizes of the protein standard refer to IgM hexamer (1236 kDa), IgM pentamer (1048 kDa), Apoferritin band 1 (720 kDa), Apoferritin band 2 (480 kDa) and B-phycoerythrin (242 kDa).

Apart from investigating its function during mouse development, we were interested in the mitochondrial topology of ATAD3 protein complexes. It was already proposed that ATAD3 forms oligomers which might span both the inner and outer mitochondrial membranes [2]. Therefore, native mitochondria from diverse human cell lines (HeLa, Jurkat E6, HEK293) and murine ES cells were isolated and subjected to Blue Native Polyacrylamide Gel Electrophoresis (BN-PAGE) and immuno-blotting. In all analyzed cells of human and murine origin, a highly similar pattern of five protein complexes containing ATAD3A/Atad3 was found (Fig. 6A). The largest complex of about 800 to 900 kDa is the most abundant one. The smaller sub-complexes have estimated molecular weights of about 720, 600, 480 and 240 kDa (Fig. 6B). Since the proteins maintain in their native conformation during BN-PAGE, the size estimation may have an expected size error of up to 15%. Coincidentally, some of the sub-complexes co-migrate with bands of the used protein standard (Apoferritin, B-phycoerythrin). To exclude any effects from contamination of the samples, the pattern was verified in several experiments and was also seen in lanes distant from the one which contained the protein standard. Also, the variation of the detergent concentrations ranging from a detergent - protein ratio from 2∶1 to 20∶1 had no influence on the complex pattern (data not shown). Since several proteins, especially mediators of mitochondrial fission and fusion, had been proposed to interact with ATAD3A, the association of MFN1, MFN2 and DRP1 to mitochondrial protein complexes was likewise tested by BN-PAGE. Intriguingly, MFN1 is found in two complexes, which are a little bit bigger than the ATAD3A sub-complexes III and IV. However, MFN2 and DRP1 are each detected in only one native mitochondrial complex with similar size to ATAD3A sub-complex III (Figure 6B). We thus conclude that ATAD3A likely is a component of multiple mitochondrial protein complexes, which might explain previous findings pointing to a variety of ATAD3 localizations and functions in mitochondria.

## Discussion

Previous studies in human cell lines and in *Caenorhabditis elegans* showed that ATAD3 AAA+-ATPases are localized to mitochondria, where they probably are arranged in oligomers that span both mitochondrial membranes with the enzymatic domain positioned in the matrix [2], [7], [19]. It is proposed that ATAD3 is implicated in the regulation of mitochondrial and ER dynamics, as interactions with mitochondrial fission (DRP1) and fusion (mitofusins, OPA1) proteins could be proofed [2], [6], [7], [19]. The ability of ATAD3 to bind mtDNA is discussed controversially [40]–[44].

In *Caenorhabditis elegans* it was demonstrated that proper ATAD-3 function is necessary for larval development [19]. Several studies with human cancer cells pointed out a role of ATAD3A and ATAD3B in tumor progression [1], [6], [45]–[47]. In order to elucidate the relevance of ATAD3 for mammalian development and disease, we analyzed an Atad3 deficient mouse line. In these mice a loss-of-function mutation in the *Atad3* gene was established by gene trapping. The resulting mutant protein is neither able to enter the mitochondrion nor to hydrolyze ATP. This article describes the essential function of Atad3 for the progression of the first steps of post-implantation development. Atad3 deficient embryos die around E7.5 due to retardation in growth and a defective development of the trophoblast lineage. The polar trophectoderm of the blastocyst gives rise to trophoblast stem cells, which are the source of the trophoblast lineage towards the establishment of secondary trophoblast giant cells and the embryonic component of the placenta. Since a minimal trophoblast outgrowth is detectable in Atad3 deficient embryos *in?vitro*, we assume that TS cells can be established in principle, but that their maintenance as well as their subsequent differentiation is disturbed. Intrinsic and extrinsic apoptotic events are unlikely to explain the reduction of the extra-embryonic component, because neither cytochrome c release from mitochondria nor fragmentation of nuclei were noticed in mutant embryos.

During pre-implantation development from the fertilized egg to the blastocyst stage the embryo generates energy mainly by glycolysis because of the hypoxic atmosphere in the oviduct and a strongly reduced biogenesis of new mitochondria [48]–[50]. Nearly all mitochondria in the pre-implantation embryo are derived from the ooplasm [51], [52]. The first embryo-own mitochondria are generated around implantation of the blastocyst into the uterus, mainly in the trophectoderm, where 80% of the embryos total ATP is synthesized and 90% of the amino acid turnover takes place [53], [54]. This rapid generation of mitochondria is due to the rapid energy requirement and oxygen consumption of the embryo, which, at this stage, resides in an oxygenized atmosphere for the first time. Therefore, ATP production switches from glycolysis in the cytoplasm to oxidative phosphorylation (OXPHOS) in the mitochondria [55]. Increased mitochondrial activity (energy metabolism and biosynthesis) is necessary for cellular differentiation processes and is therefore mediated by the new formation and elongation of mitochondria leading to the expansion of the mitochondrial network [55]–[57].

Atad3 function is essential for early post-implantation development at a time-point when mitochondrial biogenesis, i.e. the formation of new mitochondria, and ATP production through OXPHOS are required [58], [59]. Therefore, Atad3 might play a role in the biogenesis of mitochondria in trophoblast stem cells and in differentiating trophoblasts by controlling one of the following processes: growth (swelling) of mitochondria, replication of mtDNA, transcription or translation of mtDNA encoded proteins, mitochondrial protein synthesis, folding of mtDNA encoded proteins, assembly of mitochondrial protein complexes (DRP1 oligomers, OPA1 oligomers, prohibitin oligomers, mtDNA replication machinery etc.), uptake of iron into mitochondria and incorporation of iron into complexes of the respiratory chain or cytochrome c. A loss of Atad3 function might influence mitochondrial morphology and disturb mitochondrial dynamics and also alter mitochondrial activity (inner membrane potential, oxygen consumption, ATP production).

The phenotype of a loss-of-function mutation of the *Atad3* gene in the mouse is similar to the phenotype of ATAD-3 deficiency in *Caenorhabditis elegans*, in which reduced growth of embryos and a failure of early embryonic development is observed [19]. The phenotype of the prohibitin knockout mouse is very similar to the phenotype of the *Atad3* gene trap mouse, described in this article. Prohibitin knockout mice are embryonically lethal between E3.5 and E8.5 due to a rapid retardation in growth and development [60]. In mammals as well as in *Drosophila melanogaster* and in yeast two prohibitin genes *PHB1* and *PHB2* exist. Both prohibitins are located in the inner mitochondrial membrane where they form a ring complex and mediate cristae formation [61]–[65]. Prohibitins are associated to the m-AAA protease [65], [66].

In addition to the elucidation of Atad3 function in the mouse embryo, we started analyzing the contribution of ATAD3 to native mitochondrial protein complexes in order to obtain novel clues regarding its cellular function. In mitochondria of human and murine cells, ATAD3 isoforms are organized in five different protein complexes of distinct sizes, one main complex of about 800–900 kDa and four smaller sub-complexes. Since oligomerization of this protein was proposed before [2], these five complexes might reflect definite stages of ATAD3 assembly. DRP1 and MFN2 are contained in complexes of similar sizes as compared to ATAD3 sub-complex III. The precise compositions of these native complexes, as well as their exact relations to the ATAD3A complexes need further investigation.

In the future, it will be important to analyze ATAD3 function and protein interaction network in cell types, in which a switch from glycolysis to OXPHOS occurs, i.e. in which *de novo* biogenesis of mitochondria is of tremendous importance. Since ATP production by glycolysis is postulated to be characteristic for various types of stem cells [55], [67]–[71] and because cell differentiation requires OXPHOS and expansion of the mitochondrial network [56], [57], [70], [72], [73], it will be interesting to study the role of ATAD3 in stem cells which are differentiating into their first progeny. With respect to the increasing assessment of mitochondrial function for tumorigenesis [74] and the efforts of investigating the relevance of ATAD3 up-regulation in cancer [1], [6], [45], studies in tumor stem cells might be of value, too.

## Materials and Methods

### Ethics Statement

All animal experiments were conducted in a licensed animal facility in accordance with the German law on the protection of experimental animals and were approved by local authorities of the state of Nordrhein-Westfalen (Landesamt für Natur, Umwelt und Verbraucherschutz NRW). The approval number is 8.87–50.10.31.08.158.

### Generation of an *Atad3* Gene Trap Mouse Line and Genotyping

An *Atad3* gene trap mouse line (*Atad3^GT/+^*) was generated by injection of E14TG2a.4 ES cells of the annotated clone E118D03 (obtained from the German Gene Trap Consortium, Munich, Germany) into C57BL/6N blastocysts. Chimeric offspring showing germ line transmission were backcrossed into the C57BL/6J genetic background.

Genomic DNA was isolated from tail biopsies and embryos by boiling in an appropriate volume of 50 mM NaOH for 15 minutes, followed by pH neutralization of the lysate by addition of 1/4 volume of 1 M Tris-Cl, pH 8.0. For subsequent PCR genotyping the following primers had been used: endogenous Atad3 intron 1 forward primer EF 5?-GCTGTCAACACGTAGGTCGTAGGA-3?, endogenous Atad3 intron 1 reverse primer ER 5?-CACCCGCATAAACACAAAATTGAG-3? and gene trap vector rFlipROSAbetageo(Cre)0 reverse primer VR 5?-CCAATAAACCCTCTTGCAGTTGC-3?.

For RT-PCR, first total RNA was isolated from gastrula embryos (E7.5) using the RNeasy® Micro Kit (Qiagen, Hilden, Germany). Afterwards, mRNA was reverse transcribed into cDNA with SuperScriptIII™ Reverse Transcriptase (Invitrogen, Carlsbad, USA) using an oligo(dT)_20_ primer. 3?-Atad3-specific cDNA fragments were amplified using the exon 11 forward primer 5?-CAATGGGGCGGGAGGGTGTGA-3? (starting with nt 1235 in Atad3 reference cDNA NM_179203) and the exon 16 reverse primer 5?-CGAGGAGGTGTGGGAGGCAGAGAA-3? (starting with nt 2039 in Atad3 reference cDNA NM_179203). Amplification of ribosomal protein S6 (RPS6) cDNA with the primers 5?-ATTCCTGGACTGACAGACAC-3? and 5?-GTTCTTCTTAGTGCGTTGCT-3? was performed as quantity control. All *Atad3* PCR and RT-PCR fragments were verified by sequencing.

For the amplification of cDNA fragments specific for trophoblast cell types the following primers were used: Cdx2-forward (5?-CTTTGTCAGTCCTCCGCAGT-3?), Cdx2-reverse (5?-GTCACAGGACTCAAGGGCTC-3?), Bmp4-forward (5?-ACCCAGCC TGAGTATCTGGT-3?), Bmp4-reverse (5?-ACGACCATCAGCATTCGGTT-3?), Mash2-forward (5?-GCCTACTCGTCGGAGGAAAG-3?), Mash2-reverse (5?-GCAAGGTCCG GAAGATGGAA-3?), Hand1-forward (5?-CCAAGAAGGAGAGGAGACGC-3?), Hand1-reverse (5?-CTCGGCGGGAAGTGAACATA-3?).

### Histology, Embryo Culture and Immunocytochemistry

For histological analysis of early post-implantation embryos, fixed decidua were dehydrated, embedded into paraffin, sectioned sagittally at 7 µm, dewaxed and stained with hematoxylin and eosin. For embryo outgrowth culture, E6.5 embryos were dissected from decidua in PBS containing 15% FBS, transferred onto gelatine coated coverslips and incubated in DMEM medium with Glutamax and 4.5 g/l glucose (Gibco/Invitrogen, Carlsbad, USA) containing 15% FBS (PAN, Aidenbach, Germany), 1x non-essential amino acids (PAA, Pasching, Austria), 2 mM L-glutamine, 1 mM sodium pyruvate, 100 units/ml penicillin and 0.1 mg/ml streptomycin for 3 to 4 days at 37°C and 5% CO_2_.

For immunofluorescence staining of embryo outgrowths the following primary and secondary antibodies were used: rat anti-ATAD3A raised against a N-terminal His-tagged fusion protein (FLJ 4D5 rat IgG2a, undiluted supernatant), mouse anti-Cytochrome c (6H2.B4 from BD Pharmingen; diluted 1∶100), rabbit anti-Mash2 (ab74499 from abcam, Cambridge, UK; diluted 1∶500), donkey anti-rat/FITC (Dianova/Jackson ImmunoResearch, Hamburg, Germany; diluted 1∶300), donkey anti-rabbit/Cy3 (Dianova/Jackson ImmunoResearch, Hamburg, Germany; diluted 1∶300) and goat anti-mouse/Cy3 (Dianova/Jackson ImmunoResearch, Hamburg, Germany; diluted 1∶300). Image analysis and processing of immunofluorescence staining of embryo outgrowths was performed with the confocal laser scanning microscope FluoView FV1000 (Olympus, Tokyo, Japan) and the software FV1000-ASW (Olympus, Tokyo, Japan).

### Isolation of Mitochondria and Blue Native-PAGE

HeLa CCL2 cells were obtained from ATCC (Manassas, USA) and maintained in DMEM medium with 4.5 g/l glucose (Gibco/Invitrogen, Carlsbad, USA) and 10% FBS (PAN, Aidenbach, Germany) at 37°C and 5% CO_2_. The murine Knut1 ES cell line [75] was cultured in DMEM medium with Glutamax and 4.5 g/l glucose (Gibco/Invitrogen, Carlsbad, USA) containing 15% FBS (PAN, Aidenbach, Germany), 1x non-essential amino acids (PAA, Pasching, Austria), 2 mM L-glutamine, 1 mM sodium pyruvate, 100 units/ml penicillin and 0.1 mg/ml streptomycin at 37°C and 5% CO_2_. For isolation of mitochondria, cell pellets were resuspended in ice cold homogenization buffer (20 mM HEPES pH 7.6, 220 mM mannitol, 70 mM sucrose, 1 mM EDTA, 2 mg/ml bovine serum albumin, 0.5 mM PMSF) and homogenized two times by 30 strokes in a glass potter. After each homogenization cell debris was removed by centrifugation at 4000 rpm for 5 minutes. The mitochondria containing supernatant was centrifuged at 14000 rpm for 20 minutes. The pelleted mitochondria were washed once in mito buffer (20 mM HEPES pH 7.6, 220 mM mannitol, 70 mM sucrose, 1 mM EDTA, 0.5 mM PMSF) at 14000 rpm for 20 minutes, the washed mitochondria were resuspended in mito buffer. For Blue Native Polyacrylamid Gel Electrophoresis (BN-PAGE) 100 µg digitonin and 25 µg Coomassie G-250 were added to 10 µg of mitochondrial proteins. The proteins were separated under native conditions in a mini gel (polyacrylamide concentration gradient from 3% to 12%) at 150 V for 2.5 hours. Afterwards the proteins were blotted onto a PVDF membrane (Invitrogen, Carlsbad, USA). Before immune detection the blotted proteins were fixed to the membrane by incubation with 8% acidic acid for 15 minutes. For immune detection on Western blots the following primary and secondary antibodies were used: rat anti-ATAD3A (FLJ 4D5, supernatant, diluted 1∶150) rabbit anti-ATAD3A (D01 from Abnova, Taipei, Taiwan; diluted 1∶200), rabbit anti-Drp1 (H-300 from Santa Cruz Biotechnology, Santa Cruz, USA; diluted 1∶200), mouse anti-Mfn1 (M04 from Abnova, Taipei, Taiwan; diluted 1∶200), rabbit anti-Mfn2 (H-68 from Santa Cruz Biotechnology, Santa Cruz, USA; diluted 1∶200), donkey anti-rat/HRP (Dianova/Jackson ImmunoResearch, Hamburg, Germany; diluted 1∶10000), goat anti-rabbit/HRP (Dianova/Jackson ImmunoResearch, Hamburg, Germany; diluted 1∶10000), donkey anti-mouse/HRP (Dianova/Jackson ImmunoResearch, Hamburg, Germany; diluted 1∶10000), donkey anti-goat/HRP (Dianova/Jackson ImmunoResearch, Hamburg, Germany; diluted 1∶10000).

## References

[1] Schaffrik M, Mack B, Matthias C, Rauch J, Gires O (2006) Molecular characterization of the tumor-associated antigen AAA-TOB3. Cell Mol Life Sci 63: 2162–2174. 16909202 10.1007/s00018-006-6200-x PMC11135981

[2] Gilquin B, Taillebourg E, Cherradi N, Hubstenberger A, Gay O, et al (2010) The AAA+ ATPase ATAD3A controls mitochondrial dynamics at the interface of the inner and outer membranes. Mol Cell Biol 30: 1984–96. 20154147 10.1128/MCB.00007-10 PMC2849464

[3] Neuwald AF, Aravind L, Spouge JL, Koonin EV (1999) AAA+: A class of chaperone-like ATPases associated with the assembly, operation, and disassembly of protein complexes. Genome Res 9: 27–43. 9927482

[4] Langer T, Käser M, Klanner C, Leonhard K (2001) AAA proteases of mitochondria: quality control of membrane proteins and regulatory functions during mitochondrial biogenesis. Biochem Soc Trans 29: 431–6. 11498003 10.1042/bst0290431

[5] Hanson PI, Whiteheart SW (2005) AAA+ proteins: have engine, will work. Nat Rev Mol Cell Biol 6: 519–29. 16072036 10.1038/nrm1684

[6] Fang HY, Chang CL, Hsu SH, Huang CY, Chiang SF, et al (2010) ATPase family AAA domain-containing 3A is a novel anti-apoptotic factor in lung adenocarcinoma cells. J Cell Sci 123: 1171–80. 20332122 10.1242/jcs.062034

[7] Da Cruz S, Xenarios I, Langridge J, Vilbois F, Parone PA, et al (2003) Proteomic analysis of the mouse liver mitochondrial inner membrane. J Biol Chem 278: 41566–41571. 12865426 10.1074/jbc.M304940200

[8] Hubstenberger A, Merle N, Charton R, Brandolin G, Rousseau D (2010) Topological analysis of ATAD3A insertion in purified human mitochondria. J Bioenerg Biomembr 42: 143–50. 20349121 10.1007/s10863-010-9269-8

[9] Lenzen CU, Steinmann D, Whiteheart SW, Weis WI (1998) Crystal structure of the hexamerization domain of N-ethylmaleimide-sensitive fusion protein. Cell 94: 525–36. Nucleic Acids Res 37: 5701–13. 10.1016/s0092-8674(00)81593-7 9727495

[10] Zhang X, Shaw A, Bates PA, Newman RH, Gowen B, et al (2000) Structure of the AAA ATPase p97. Mol Cell 6: 1473–84. 11163219 10.1016/s1097-2765(00)00143-x

[11] Detmer SA, Chan DC (2007) Functions and dysfunctions of mitochondrial dynamics. Nat Rev Mol Cell Biol 8: 870–9. 17928812 10.1038/nrm2275

[12] Benard G, Karbowski M (2009) Mitochondrial fusion and division: Regulation and role in cell viability. Semin Cell Dev Biol 20: 365–74. 19530306 10.1016/j.semcdb.2008.12.012 PMC2768568

[13] Liesa M, Palacín M, Zorzano A (2009) Mitochondrial dynamics in mammalian health and disease. Physiol Rev 89: 799–845. 19584314 10.1152/physrev.00030.2008

[14] Westermann B (2010) Mitochondrial fusion and fission in cell life and death. Nat Rev Mol Cell Biol 11: 872–84. 21102612 10.1038/nrm3013

[15] Mancini M, Anderson BO, Caldwell E, Sedghinasab M, Paty PB, et al (1997) Mitochondrial proliferation and paradoxical membrane depolarization during terminal differentiation and apoptosis in a human colon carcinoma cell line. J Cell Biol 138: 449–69. 9230085 10.1083/jcb.138.2.449 PMC2138196

[16] De Vos K, Goossens V, Boone E, Vercammen D, Vancompernolle K, et al (1998) The 55-kDa tumor necrosis factor receptor induces clustering of mitochondria through its membrane-proximal region. J Biol Chem 273: 9673–80. 9545301 10.1074/jbc.273.16.9673

[17] Frank S, Gaume B, Bergmann-Leitner ES, Leitner WW, Robert EG, et al (2001) The role of dynamin-related protein 1, a mediator of mitochondrial fission, in apoptosis. Dev Cell 1: 515–25. 11703942 10.1016/s1534-5807(01)00055-7

[18] Karbowski M, Lee YJ, Gaume B, Jeong SY, Frank S, et al (2002) Spatial and temporal association of Bax with mitochondrial fission sites, Drp1, and Mfn2 during apoptosis. J Cell Biol 159: 931–8. 12499352 10.1083/jcb.200209124 PMC2173996

[19] Hoffmann M, Bellance N, Rossignol R, Koopman WJ, Willems PH, et al (2009) C. elegans ATAD-3 is essential for mitochondrial activity and development. PLoS One 4: e7644. 19888333 10.1371/journal.pone.0007644 PMC2765634

[20] James DI, Parone PA, Mattenberger Y, Martinou JC (2003) hFis1, a novel component of the mammalian mitochondrial fission machinery. J Biol Chem 278: 36373–9. 12783892 10.1074/jbc.M303758200

[21] Yoon Y, Krueger EW, Oswald BJ, McNiven MA (2003) The mitochondrial protein hFis1 regulates mitochondrial fission in mammalian cells through an interaction with the dynamin-like protein DLP1. Mol Cell Biol 23: 5409–20. 12861026 10.1128/MCB.23.15.5409-5420.2003 PMC165727

[22] Smirnova E, Griparic L, Shurland DL, van der Bliek AM (2001) Dynamin-related protein Drp1 is required for mitochondrial division in mammalian cells. Mol Biol Cell. 12: 2245–56. 10.1091/mbc.12.8.2245 PMC58592 11514614

[23] Yoon Y, Pitts KR, McNiven MA (2001) Mammalian dynamin-like protein DLP1 tubulates membranes. Mol Biol Cell 12: 2894–905. 11553726 10.1091/mbc.12.9.2894 PMC59722

[24] Jagasia R, Grote P, Westermann B, Conradt B (2005) DRP-1-mediated mitochondrial fragmentation during EGL-1-induced cell death in C. elegans. Nature 433: 754–60. 15716954 10.1038/nature03316

[25] Eura Y, Ishihara N, Yokota S, Mihara K (2003) Two mitofusin proteins, mammalian homologues of FZO, with distinct functions are both required for mitochondrial fusion. J Biochem. 134: 333–44. 10.1093/jb/mvg150 14561718

[26] Santel A, Frank S, Gaume B, Herrler M, Youle RJ, et al (2003) Mitofusin-1 protein is a generally expressed mediator of mitochondrial fusion in mammalian cells. J Cell Sci 116: 2763–74. 12759376 10.1242/jcs.00479

[27] Detmer SA, Chan DC (2007) Complementation between mouse Mfn1 and Mfn2 protects mitochondrial fusion defects caused by CMT2A disease mutations. J Cell Biol. 176: 405–14. 10.1083/jcb.200611080 PMC2063976 17296794

[28] Song Z, Ghochani M, McCaffery JM, Frey TG, Chan DC (2009) Mitofusins and OPA1 mediate sequential steps in mitochondrial membrane fusion. Mol Biol Cell 20: 3525–32. 19477917 10.1091/mbc.E09-03-0252 PMC2719570

[29] de Brito OM, Scorrano L (2008) Mitofusin 2 tethers endoplasmic reticulum to mitochondria. Nature 456: 605–10. 19052620 10.1038/nature07534

[30] de Brito OM, Scorrano L (2009) Mitofusin-2 regulates mitochondrial and endoplasmic reticulum morphology and tethering: the role of Ras. Mitochondrion 9: 222–6. 19269351 10.1016/j.mito.2009.02.005

[31] de Brito OM, Scorrano L (2010) An intimate liaison: spatial organization of the endoplasmic reticulum-mitochondria relationship. EMBO J 29: 2715–23. 20717141 10.1038/emboj.2010.177 PMC2924651

[32] Griparic L, van der Wel NN, Orozco IJ, Peters PJ, van der Bliek AM (2004) Loss of the intermembrane space protein Mgm1/OPA1 induces swelling and localized constrictions along the lengths of mitochondria. J Biol Chem 279: 18792–8. 14970223 10.1074/jbc.M400920200

[33] Cipolat S, Martins de Brito O, Dal Zilio B, Scorrano L (2004) OPA1 requires mitofusin 1 to promote mitochondrial fusion. Proc Natl Acad Sci U S A 101: 15927–32. 15509649 10.1073/pnas.0407043101 PMC528769

[34] Frezza C, Cipolat S, Martins de Brito O, Micaroni M, Beznoussenko GV, et al (2006) OPA1 controls apoptotic cristae remodeling independently from mitochondrial fusion. Cell. 126: 177–89. 10.1016/j.cell.2006.06.025 16839885

[35] Song Z, Chen H, Fiket M, Alexander C, Chan DC (2007) OPA1 processing controls mitochondrial fusion and is regulated by mRNA splicing, membrane potential, and Yme1L. J Cell Biol 178: 749–55. 17709429 10.1083/jcb.200704110 PMC2064540

[36] Ehses S, Raschke I, Mancuso G, Bernacchia A, Geimer S, et al (2009) Regulation of OPA1 processing and mitochondrial fusion by m-AAA protease isoenzymes and OMA1. J Cell Biol 187: 1023–36. 20038678 10.1083/jcb.200906084 PMC2806285

[37] Susin SA, Lorenzo HK, Zamzami N, Marzo I, Snow BE, et al (1999) Molecular characterization of mitochondrial apoptosis-inducing factor. Nature 397: 441–6. 9989411 10.1038/17135

[38] Artus C, Boujrad H, Bouharrour A, Brunelle MN, Hoos S, et al (2010) AIF promotes chromatinolysis and caspase-independent programmed necrosis by interacting with histone H2AX. EMBO J 29: 1585–99. 20360685 10.1038/emboj.2010.43 PMC2876946

[39] Gilquin B, Cannon BR, Hubstenberger A, Moulouel B, Falk E, et al (2010) The calcium-dependent interaction between S100B and the mitochondrial AAA ATPase ATAD3A and the role of this complex in the cytoplasmic processing of ATAD3A. Mol Cell Biol 30: 2724–36. 20351179 10.1128/MCB.01468-09 PMC2876520

[40] He J, Mao CC, Reyes A, Sembongi H, Di Re M, et al (2007) The AAA+ protein ATAD3 has displacement loop binding properties and is involved in mitochondrial nucleoid organization. J Cell Biol 176: 141–6. 17210950 10.1083/jcb.200609158 PMC2063933

[41] Holt IJ, He J, Mao CC, Boyd-Kirkup JD, Martinsson P, et al (2007) Mammalian mitochondrial nucleoids: organizing an independently minded genome. Mitochondrion 7: 311–21. 17698423 10.1016/j.mito.2007.06.004

[42] Bogenhagen DF, Rousseau D, Burke S (2008) The layered structure of human mitochondrial DNA nucleoids. J Biol Chem 283: 3665–3675. 18063578 10.1074/jbc.M708444200

[43] Di Re M, Sembongi H, He J, Reyes A, Yasukawa T, et al. (2009) The accessory subunit of mitochondrial DNA polymerase gamma determines the DNA content of mitochondrial nucleoids in human cultured cells. Nucleic Acid Res 37: 5701–13. Available: http://www.ncbi.nlm.nih.gov/pubmed?term=The accessory subunit of mitochondrial DNA polymerase gamma determines the DNA content of mitochondrial nucleoids in human cultured cells. 10.1093/nar/gkp614 PMC2761280 19625489

[44] He J, Cooper HM, Reyes A, Di Re M, Sembongi H, et al (2012) Mitochondrial nucleoid interacting proteins support mitochondrial protein synthesis. Nucleic Acids Res 40: 6109–21. 22453275 10.1093/nar/gks266 PMC3401451

[45] Geuijen CA, Bijl N, Smit RC, Cox F, Throsby M, et al (2005) A proteomic approach to tumour target identification using phage display, affinity purification and mass spectrometry. Eur J Cancer 41: 178–87. 15618003 10.1016/j.ejca.2004.10.008

[46] Hubstenberger A, Labourdette G, Baudier J, Rousseau D (2008) ATAD 3A and ATAD 3B are distal 1p-located genes differentially expressed in human glioma cell lines and present in vitro anti-oncogenic and chemoresistant properties. Exp Cell Res 314: 2870–83. 18639545 10.1016/j.yexcr.2008.06.017

[47] Merle N, Féraud O, Gilquin B, Hubstenberger A, Kieffer-Jacquinot S, et al (2012) ATAD3B is a human embryonic stem cell specific mitochondrial protein, re-expressed in cancer cells, that functions as dominant negative for the ubiquitous ATAD3A. Mitochondrion 12: 441–8. 22664726 10.1016/j.mito.2012.05.005

[48] Brown GC (1992) Control of respiration and ATP synthesis in mammalian mitochondria and cells. Biochem. J. 284: 1–13. 10.1042/bj2840001 PMC1132689 1599389

[49] Fisher B, Bavister BD (1993) Oxygen tension in the oviduct and uterus of rhesus monkeys, hamsters and rabbits. J. Reprod. Fertil. 99: 673–679. 10.1530/jrf.0.0990673 8107053

[50] Van Blerkom J, Davis P, Mathwig V, Alexander S (2002) Domains of high-polarized and low-polarized mitochondria may occur in mouse and human oocytes and early embryos. Human Reproduction 17: 393–406. 11821285 10.1093/humrep/17.2.393

[51] Kaneda H, Hayashi J, Takahama S, Taya C, Lindahl KF, et al (1995) Elimination of paternal mitochondrial DNA in intraspecific crosses during early mouse embryogenesis. Proceedings of the National Academy of Sciences of the United States of America 92: 4542–4546. 7753839 10.1073/pnas.92.10.4542 PMC41980

[52] Cummins JM (2000) Fertilization and elimination of the paternal mitochondrial genome. Hum Reprod 15: 92–101. 11041517 10.1093/humrep/15.suppl_2.92

[53] Houghton FD (2006) Energy metabolism of the inner cell mass and trophectoderm of the mouse blastocyst. Differentiation 74: 11–18. 16466396 10.1111/j.1432-0436.2006.00052.x

[54] Van Blerkom J (2008) Mitochondria as regulatory forces in oocytes, preimplantation embryos and stem cells. Reprod Biomed Online 16: 553–569. 18413065 10.1016/s1472-6483(10)60463-4

[55] Cho YM, Kwon S, Pak YK, Seol HW, Choi YM, et al (2006) Dynamic changes in mitochondrial biogenesis and antioxidant enzymes during the spontaneous differentiation of human embryonic stem cells. Biochemical and Biophysical Research Communications 348: 1472–1478. 16920071 10.1016/j.bbrc.2006.08.020

[56] St John JC, Ramalho-Santos J, Gray HL, Petrosko P, Rawe VY, et al (2005) The expression of mitochondrial DNA transcription factors during early cardiomyocyte in vitro differentiation from human embryonic stem cells. Cloning Stem Cells. 7: 141–53. 10.1089/clo.2005.7.141 16176124

[57] Chung S, Dzeja PP, Faustino RS, Perez-Terzic C, Behfar A, et al (2007) Mitochondrial oxidative metabolism is required for the cardiac differentiation of stem cells. Nature Clinical Practice Cardiovascular Medicine 4: S60–S67. 10.1038/ncpcardio0766 PMC3232050 17230217

[58] Dumollard R, Duchen M, Sardet C (2006) Calcium signals and mitochondria at fertilisation. Semin Cell Dev Biol 17: 314–323. 16574440 10.1016/j.semcdb.2006.02.009

[59] Facucho-Oliveira JM, Alderson J, Spikings EC, Egginton S, St John JC (2007) Mitochondrial DNA replication during differentiation of murine embryonic stem cells. J Cell Sci 120: 4025–34. 17971411 10.1242/jcs.016972

[60] He B, Feng Q, Mukherjee A, Lonard DM, DeMayo FJ, et al (2007) A repressive role for prohibitin in estrogen signaling. Molecular Endocrinology 22: 344–360. 17932104 10.1210/me.2007-0400 PMC2234581

[61] Coates PJ, Jamieson DJ, Smart K, Prescott AR, Hall PA (1997) The prohibitin family of mitochondrial proteins regulate replicative lifespan. Curr Biol 7: 607–10. 9259555 10.1016/s0960-9822(06)00261-2

[62] Nijtmans LG, de Jong L, Artal Sanz M, Coates PJ, Berden JA, et al (2000) Prohibitins act as a membrane-bound chaperone for the stabilization of mitochondrial proteins. EMBO J 19: 2444–51. 10835343 10.1093/emboj/19.11.2444 PMC212747

[63] Tatsuta T, Model K (2005) Langer (2005) Formation of membrane-bound ring complexes by prohibitins in mitochondria. Mol Biol Cell 16: 248–59. 15525670 10.1091/mbc.E04-09-0807 PMC539169

[64] Merkwirth C, Dargazanli S, Tatsuta T, Geimer S, Löwer B, et al (2008) Prohibitins control cell proliferation and apoptosis by regulating OPA1-dependent cristae morphogenesis in mitochondria. Genes Dev 22: 476–88. 18281461 10.1101/gad.460708 PMC2238669

[65] Merkwirth C, Langer T (2009) Prohibitin function within mitochondria: essential roles for cell proliferation and cristae morphogenesis. Biochim Biophys Acta 1793: 27–32. 18558096 10.1016/j.bbamcr.2008.05.013

[66] Steglich G, Neupert W, Langer T (1999) Prohibitins regulate membrane protein degradation by the m-AAA protease in mitochondria. Mol Cell Biol 19: 3435–42. 10207067 10.1128/mcb.19.5.3435 PMC84136

[67] Piccoli C, Ria R, Scrima R, Cela O, D’Aprile A, et al (2005) Characterization of mitochondrial and extra-mitochondrial oxygen consuming reactions in human hematopoietic stem cells. Novel evidence of the occurrence of NAD(P)H oxidase activity. Journal of Biological Chemistry 280: 26467–26476. 15883163 10.1074/jbc.M500047200

[68] Bavister BD (2006) The mitochondrial contribution to stem cell biology. Reproduction, Fertility and Development 18: 829–838. 10.1071/rd06111 17147931

[69] Lonergan T, Brenner C, Bavister B (2006) Differentiation-related changes in mitochondrial properties as indicators of stem cell competence. Journal of Cellular Physiology 208: 149–153. 16575916 10.1002/jcp.20641

[70] Kondoh H, Lleonart ME, Nakashima Y, Yokode M, Tanaka M, et al (2007) A high glycolytic flux supports the proliferative potential of murine embryonic stem cells. Antioxidants & Redox Signalling 9: 293–299. 10.1089/ars.2006.1467 17184172

[71] Chen CT, Shih YR, Kuo TK, Lee OK, Wei YH (2008) Coordinated changes of mitochondrial biogenesis and antioxidant enzymes during osteogenic differentiation of human mesenchymal stem cells. Stem Cells 26: 960–968. 18218821 10.1634/stemcells.2007-0509

[72] Facucho-Oliveira JM, St John JC (2009) The relationship between pluripotency and mitochondrial DNA proliferation during early embryo development and embryonic stem cell differentiation. Stem Cell Rev 5: 140–58. 19521804 10.1007/s12015-009-9058-0

[73] Mandal S, Lindgren AG, Srivastava AS, Clark AT, Banerjee U (2011) Mitochondrial function controls proliferation and early differentiation potential of embryonic stem cells. Stem Cells. 29: 486–95. 10.1002/stem.590 PMC4374603 21425411

[74] Gogvadze V, Orrenius S, Zhivotovsky B (2008) Mitochondria in cancer cells: what is so special about them? Trends Cell Biol 18: 165–73. 18296052 10.1016/j.tcb.2008.01.006

[75] Peitz M, Jäger R, Patsch C, Jäger A, Egert A, et al (2007) Enhanced purification of cell-permeant Cre and germline transmission after transduction into mouse embryonic stem cells. Genesis 45: 508–517. 17661398 10.1002/dvg.20321

